# Management of Hyperparasitaemia in Severe Malaria: A Sea Change With the Availability of Artesunate

**DOI:** 10.7759/cureus.56894

**Published:** 2024-03-25

**Authors:** Bipul K Das, Himesh Barman, Rashna Dass Hazarika, Ajit Chhetri

**Affiliations:** 1 Paediatrics, All India Institute of Medical Sciences, Guwahati, IND; 2 Paediatrics, North Eastern Indira Gandhi Regional Institute of Health and Medical Sciences, Shillong, IND; 3 Paediatrics and Neonatology, PA Sangma International Medical College and Hospital, Baridua, IND; 4 Paediatrics, Medica North Bengal Clinic, Siliguri, IND

**Keywords:** children, artesunate, exchange blood transfusion, hyperparasitaemia, malaria

## Abstract

Objective: This study aims to study the efficacy of exchange transfusion in complicated malaria with hyperparasitaemia.

Method: This is a retrospective case-control study conducted in a tertiary care referral hospital in northeastern India. A retrospective chart review was done, and 34 children with hyperparasitaemia were identified. Of these, 16 children received only antimalarial chemotherapy, and 18 received adjunct exchange blood transfusion (EBT). The data was analyzed for survival benefit as the main outcome measure.

Results: The subjects in each of the groups were comparable in terms of age, sex distribution, and mean haemoglobin at presentation. The patients treated with EBT were more ill than those who did not receive EBT; they met a greater number of WHO criteria for severe malaria (2.94 ± 1.16 vs. 1.81 ± 0.83; p=0.002) and had higher levels of parasitaemia (23.96 ± 17.55 vs. 16.14 ± 6.89; p=0.0007). The mean volume exchanged was 44.88 ± 20.49 ml/kg, against a target of 70 ml/kg due to the unavailability of fresh, compatible whole blood. There was no difference in mortality in the exchange transfusion group compared to the chemotherapy alone group (22.22% vs. 31.25%; p=0.83).

Conclusions: In resource-limited areas, lower volumes of fresh whole blood for exchange transfusion can reduce hyperparasitaemia to a significant degree, and this may give some mortality benefit in very sick cases. Artesunate, by virtue of its rapid action, is capable of reducing hyperparasitaemia and may question the very place of blood transfusion in complicated malaria. It may also be worthwhile to accumulate more data comparing EBT against artesunate instead of quinine.

## Introduction

Malaria is a parasitic disease caused by four species of malarial parasites. Of these *Plasmodium falciparum* causes, the most serious disease has the highest fatality rate [1]. In 2022, about 249 million people were infected by malaria worldwide, with an estimated death rate of approximately 608,000. Infants and children under five years of age are part of the vulnerable population who are at risk of developing severe malaria [1]. Anti-parasitic chemotherapy has been the mainstay of treatment. This may, at times, fail due to an overwhelming parasitic load or a lack of time for the drug to act before the disease is fatal. Hyperparasitaemia in falciparum malaria is a special concern because these patients are at higher risk of mortality and higher treatment failure rates, and they are also a source of potential new drug resistance. Hyperparasitaemia is one of the most common reasons for which patients receive treatment for severe malaria. Furthermore, hyperparasitaemia is known to be an important marker of severe malaria [2]. Exchange transfusion was first done in 1974 for this indication, and since then, it has emerged in the current literature as an adjunct emergency therapy in cases of complicated malaria with hyperparasitaemia [3]. There have been many anecdotal reports and several series claiming the benefits of exchange blood transfusion (EBT) in severe malaria but no controlled trials, and there is no consensus on whether EBT reduces mortality or how it actually works [1]. It is believed to reduce the parasite load, remove toxic substances, reduce microcirculatory sludging, and increase the oxygen-carrying capacity of the blood [4-6]. These studies have come mostly from developed countries and Thailand, and the place of EBT in developing countries has been questioned [6,7]. Our experience addresses the issue of the practicability of the procedure in a resource-limited setting and its efficacy under such circumstances. Moreover, to the best of our knowledge and belief, this is the first comparative study using artesunate as the primary antimalarial drug.

## Materials and methods

This is a retrospective case-control study conducted in a tertiary care hospital in a resource-poor region in northeast India. The discharge and death summaries of cases admitted to the paediatric intensive care unit and paediatric general ward over a period of three years, from September 2006 to August 2009, were reviewed to identify the cases of complicated malaria with hyerparasitaemia. The number of parasitised red blood cells per 100 red cells in peripheral blood smear was the parasite index (PI), which was used for malaria load estimation. Hyperparasitaemia was defined by a parasite index of >5% [2]. Positive rapid diagnostic tests for malaria and positive peripheral blood smears with both thick and thin films in children with clinical symptoms consistent with malaria were used for confirmation of the diagnosis of malaria in children. A total of 162 cases of proven malaria were found, of which 34 had a PI greater than 5%. The patient files were retrieved from the medical record department of the institute. The case files of these cases were reviewed retrospectively, and the relevant data were collected and analyzed. Out of these, 18 (52.9%) cases were treated with adjunct EBT and 16 (47%) with chemotherapy and supportive measures only. In all the cases who received EBT, an exchange volume of 70 ml/kg was targeted, but in cases of the unavailability of enough blood, the procedure was carried out with lesser amounts of blood volume as made available from the blood bank. The procedures were carried out by a manual push-pull technique via central venous access with an aliquot size of 20 to 25 ml. Two to four units of fresh whole blood were available per patient, which amounted to 15 to 87 ml/kg, depending upon the body weight of the patient. Survival was taken as the primary outcome variable for the purpose of the analysis. Secondary outcome variables included rapidity of initial drop in parasitaemia, time of resolution of hyperparasitaemia, time of parasite clearance from peripheral blood smear, and duration of hospital stay. Statistical analysis was done by Microsoft Excel (Microsoft Corporation, Redmond, WA, USA) and OpenEPi software version 2.3.1 (www.OpenEpi.com).

## Results

A total of 34 cases of complicated malaria with hyperparasitaemia were encountered. Twenty-eight children were started on quinine and six on artesunate as the first line of antimalarial. A switch to artesunate was done in those patients on quinine who were detected to have hyperparasitaemia. Sixteen children were treated with chemotherapy alone, and the remaining 18 also underwent adjunct EBT. The modality of treatment was decided by the attending clinician and was based on the severity of the disease in a hyperparasitemic child. All of these cases were managed in the pediatric intensive care unit and received optimal supportive care in the form of optimal fluid and electrolyte management to maintain normothermia, normoglycemia, seizure control, nutritional management, and appropriate nursing care for sick children in the pediatric intensive care unit. Although the subjects from each of the groups were comparable in terms of age, sex distribution, and mean haemoglobin at presentation (Table 1), the patients treated with EBT were more ill than those patients who did not receive transfusions; the EBT group patients met a greater number of WHO criteria for severe malaria (2.94 ± 1.16 vs. 1.81 ± 0.83; p=0.002) and had higher levels of parasitaemia (23.96 ± 17.55 vs. 16.14 ± 6.89; p=0.0007). The overall PI in the 34 patients ranged from 6% to 70% (mean: 20.27%), whereas the PI ranged from 8.2% to 70% in the exchange group and 6% to 32% in the control group.

**Table 1:** Baseline data of the two groups

Mean of variables	Adjunct exchange group (n=18)	Antimalarial chemotherapy only group (n=16)	p-value
Mean peak parasitaemia (%)	23. 96 + 17.55	16.14 + 6.89	0.0007
Mean admission haemoglobin (gm/dl)	8.88 + 2.76	8.84 + 2.61	0.86
Mean age (years)	9.58 + 5.6	8.81 + 5.5	0.89
Sex (male:female)	10:8	13:3	0.11
Mean number of patients with WHO criteria of severe malaria	2.94 + 1.16	1.81 + 0.83	0.002

The mean volume exchanged was 44.88 ± 20.49 ml/kg. A total of 51 units (46 for EBT and five for transfusion) (Table 2) of blood were needed in the EBT group, whereas the artesunate group required only four units of blood. The mean lag time from admission to initiation of exchange transfusion was 6.7 ± 2.7 hours, and the exchange transfusions were done over half an hour to 2.5 hours, with a mean of 1.86 ± 0.70 hours. One patient had hypotension during the procedure, which was managed by fluids and inotropes without abandoning the procedure; the rest all tolerated the procedure well.

**Table 2:** Comparison of both groups in terms of outcome variables

Variables	Adjunct exchange group	Antimalarial chemotherapy only group	p-value
Primary outcome			
Survival	4 out of 18	5 out of 16	p=0.83
Corrected survival	3 out of 17	1 out of 12	p=0.93
Secondary outcome			
Mean hours taken for hyperparacytaemia clearance	12.66 (95% CI: 8.33-16.98)	19.09 (95% CI: 14.12-24.05)	p=0.06
Mean hours taken for parasite clearance	24.70 (95% CI: 17.00-32.29)	29.64 (95% CI: 22.02-37.25)	p=0.31
Net change in parasite index	18.97 (95% CI: 9.47-28.4)	4.07 (95% CI: 2.45-5.68)	p=0.01
Duration stay of survivors in days	9.57 (95% CI: 5.88-13.25)	8.54 (95% CI: 3.62-13.45)	p=0.77
Blood units consumed	46 (exchange) + 5 (transfusion)	4 (transfusion)	

Details of the drugs used are given in Table 3. There was apparently decreased mortality in the exchange transfusion group compared to the chemotherapy-only group (22.22% vs. 31.25%). However, the difference was statistically insignificant (p=0.83). One child in the exchange transfusion group died at 35 days of admission, the cause of death being a nosocomial infection that was not related to malaria per se. On the other hand, two children in the chemotherapy alone group had an increase in parasitaemia after admission, and the condition deteriorated fast and died even before the exchange transfusion could be planned (blood report of hyperparasitaemia received at the terminal stage). Two more children in the chemotherapy-only group were initially planned for exchange transfusions, but the children died before the procedure could be undertaken. A corrected mortality analysis was done by excluding these children from their respective groups, which showed increased mortality in the exchange transfusion group (17.6% vs. 6%). However, this difference was also statistically insignificant (p=0.86). Therefore, there was no difference in primary outcome benefit observed between the two groups. However, it was observed that there was a significantly more precipitous drop in PI following exchange transfusion compared to chemotherapy alone. The net change in PI following exchange transfusion was 18.97% (95% CI: 9.47-28.4) compared to a drop of 4.07% (95% CI: 2.45-5.68) over the first six hours in the chemotherapy-only group (p=0.01). There was also a trend towards early resolution of hyperparasitaemia (12.66; 95% CI: 8.33-16.98 vs. 19.09; 95% CI: 14.12-24.05 hours, p=0.06) in the exchange group, but this did not translate into statistical significance. The mean parasite clearance time from peripheral blood smear was similar in both groups (24.70; 95% CI: 17.00-32.29 vs. 29.64; 95% CI: 22.02-37.25 hours, p=0.31). The mode of treatment did not affect the duration of hospital stay in survivors (9.36 days; 95% CI: 5.88-13.25 vs. 8.54 days; 95% CI: 3.62-13.45, p=0.77).

**Table 3:** Details of the cases and modality of treatment used A: artesunate, Q: quinine, C: clindamycin, D: doxycycline, EBT: exchange blood transfusion

Sl no.	Name	Age	Sex	Hemoglobin	Pre-exchange parasite index	Post-exchange parasite index	Net change in parasite index	Units of blood used for EBT	ml/ kg of blood used	Time taken (procedure)	Hyperparasitaemia clearance time (hrs)	Para site clearance time (hrs)	Drug used	Outcome	Fever clearance in survivors (days )	Duration of stay of survivors in days
E 1	WN	3	F	6.7	8.2	0	8.2	2	56	2.5	6	6	A, Q	Discharged	8	10
E 2	PM	6	M	6.6	7.8	6	1.8	2	39	2.2	18	24	A, Q	Discharged	2	5
E 3	TGM	13	F	11.5	14.5	7.0	7.7	3	50	1.4	30	42	A, C	Discharged	1	8
E 4	AS	13	F	11.5	17.5	9.5	8.0	3	47	2.0	24	42	A, D	Discharged	3	5
E 5	NL	6	M	9.5	35	8.2	26.8	3	65	2.2	12	24	A C	Discharged	20	23
E 6	RKM	12	F	10.7	21	9	12	3	40	1.5	24	42	A, Q, D	Discharged	2	6
E 7	AD	16	F	10	29	20.5	9	3	35	2	30	48	A, C	Expired		
E 8	WI	2	M	4.2	52	9.2	42.8	2	75	1.5	12	42	A, C	Discharged	5	6
E 9	RTM	14	F	4.3	8.6	3	5.6	3	26	1.2	6	12	A, Q	Expired		
E 10	LS	11	F	8.1	70	1	69	2	23	0.5	6	12	Q, A, C	Expired		
E 11	KS	8	M	9.1	20	1	19	2	28	1	6	24	Q, A, D	Discharged	7	8
E 12	BM	7	M	7.5	21	7	14	2	38	3	12	18	A, Q, D	Discharged	4	13
E 13	RS	18	M	15	15.5	0.8	14.7	3	21	2	6	6	Q, A	Expired		
E 14	AS	17	F	12	8.2	0	8.2	4	31	1.6	6	12	Q, A	Discharged	2	5
E 15	PM	17	M	9	8.4	0.3	8.1	2	15	1	6	30	A, Q, C	Discharged	2	9
E 16	PN	3.5	M	9.1	12.6	5.4	7.2	3	87	2.5	12	18	A, C	Discharged	4	4
E 17	AS	4	M	9	42	0.5	41.5	2	62	2.5	6	18	A, Q	Discharged	1	24
E 18	SK	2	M	6	40	2	38	2	70	3	6	12	A, Q	Discharged	5	8
Sl no.	Name	Age	Sex	Hemoglobin	Peak parasite index	Parasite index at 6 hrs	Net change in parasite index				Hyperparasitaemia clearence (hrs)	Parasite clearance time (hrs)	Drugs	Outcome	Fever clearance in survivors (days)	Duration of stay of survivors in days
D 1	TS	12	M	12.0	14.5	6.8	7.7	Conservative group: antimalarial drugs only			30	36	Q, A	Discharged	5	8
D 2	VK	17	M	9.5	28	-	-				-	-	Q, A	Expired		
D 3	LS	3	M	10.5	18	14	4				18	24	Q, A	Discharged	2	8
D 4	SS	17	M	5.9	32	-	-				-	-	Q, A	Expired		
D 5	IT	2.5	M	9.2	13	10	3				18	30	Q, A	Discharged	13	20
D6	DB	6	F	10.3	15.5	13.6	1.9				24	42	Q, A	Discharged	21	25
D7	AP	5	M	7.8	14.7	7.9	6.8				12	18	Q, A	Discharged	2	5
D8	PL	9	M	13.2	12.8	10.5	2.3				24	30	Q, A	Discharged	5	6
D9	LM	3.5	M	9.5	20	-	-				-	-	Q, A	Expired		
D10	MM	14	F	11.2	19	-	-				-	-	Q, A	Expired		
D 11	AK	7	F	6.8	22	15	7				12	24	A	Discharged	1	4
D 12	PK	15	M	9.9	6	5	1				36	56	Q, A	Discharged	2	6
D 13	JN	9	M	5.4	11.8	-	-						Q, A	Expired	-	
D 14	LK	2.5	M	4.1	12.5	6.2	6.3				12	24	Q, A	Discharged	1	2
D 15	SKM	15	M	11.5	10.4	8.4	2				12	24	Q, A	Discharged	2	2
D 16	DS	3.5	M	6.0	8	5.2	2.8				12	18	A	Discharged	6	8

## Discussion

Exchange transfusion in malaria is currently far from being a standard mode of therapy, although there is a lot of enthusiasm regarding it in the literature [2]. A number of case reports and series exist in the literature on complicated malaria with hyperparasitaemia successfully salvaged with exchange transfusion [8-18].

The reports present in the literature vary significantly with regards to when exchange transfusions are to be done and with how much blood. An adult literature search revealed that workers have used up to 10-15 units of blood at various grades of parasitaemia. Srichaikul et al. (1993) did exchange transfusions in severe malaria with multisystem involvement in parasitaemia ranging from 0.3-90%. They used 10-15 units of blood and recommended that the minimum amount of blood exchanged be 1.2 times the blood volume [9]. Pinanong (1997) did exchange transfusions in patients with >30% parasitaemia and multisystem involvement and concluded that those with ARDS and renal involvement show special benefits, and the minimum amount of blood used is 10-14 units [8]. Gulprasutdilog et al. (1999) [12] did an exchange in patients >10% and recommended that the volume for exchange be 2000 ml for 10-20% parasitaemia and 4000 ml for >20% parasitaemia. Looareesuwan et al. (1990) [11] noted that up to 10 litres of blood have been exchanged in malaria and bebesiosis, but they pointed out that partial exchange with as few as 1-2 units of blood over three to seven hours may be beneficial. Kumar et al. (2003) demonstrated the efficacy of partial exchange by documenting a fall in pre-exchange median parasitaemia from 16.4% to 4.5% post-exchange using as little as 40 ml/kg of blood.

Limited data is available in paediatrics, but the same uncertainty prevails. Deshpande et al. (2003) [16] successfully did a manual exchange in three children with >50% parasitaemia using 80 ml/kg body weight. Boctor (2005) [13] successfully did a manual exchange in one patient using 70 ml/kg of blood. Shanbag et al. (2006) [14] exchanged transfusions in four children using 160 ml/kg of blood.

On the other hand, there is equally robust evidence against the need and benefit of the procedure. Mordmüller et al. (1998) and Reisinger et al. (1992) demonstrated successful treatment of complicated malaria with hyperparasitaemia up to as high as 81% with chemotherapy alone and questioned the need for exchange transfusion for hyperparasitaemia [19,20]. A large retrospective series by Burchard et al. found that exchange transfusion did not significantly improve the unfavourable prognosis in cases of severe falciparum malaria [21]. A meta-analysis by Riddle et al. (2002), which included eight studies, concluded that EBT failed to offer survival benefits compared to antimalarial chemotherapy alone [22]. The present study also did not observe any survival benefit in the EBT group. The current study, however, is of particular importance in that it explores the scope of EBT in complicated malaria in a resource-limited setting and that the primary antimalarial used was artesunate, unlike previous workers who have used quinine. In our series, we targeted a single-volume exchange transfusion, but we could arrange only two to four units of blood per patient. In developing countries, patients’s relatives are the only potential donors. The availability of several units of fresh whole blood at a time is a big problem, especially when most of the relatives would also be anaemic, having suffered from repeated attacks of malaria themselves. So, in our series, EBT had to be done with about 15 to 87 ml/kg of blood, depending upon the body weight of the patient. The mean volume exchanged was 44.88 + 20.49 ml/kg. It is noteworthy that 44% of the patients received <40 ml/kg blood exchange, which included all four fatal cases. This may be one of the reasons for the inability to show survival benefits in the EBT group. Secondly, all the studies that showed the benefit of EBT against antimalarials alone used quinine as the antimalarial agent. Artesunate, on the other hand, is a faster malaricidal and can potentially achieve rapid parasite clearance, which is also the target of EBT. We did not find any statistically significant difference between the two groups in the time taken to bring down the PI to less than 5%. This may be one of the reasons why EBT failed to show survival benefits, unlike quinine. However, the moot point to be considered is that the patients in the EBT group were significantly sicker as per standard WHO criteria and also had a higher PI compared to the chemotherapy-only group but had a similar survival benefit as the less sick patients in the chemotherapy-only group. This hints towards a cryptic marginal advantage of EBT in the sicker patients with very high PI. The dramatic reduction of PI in the EBT group even with smaller blood volumes demonstrates that using blood volumes less than the standard recommendation of 70 ml/kg can be effective in lowering the PI and also improving the anaemic status in such sick patients.

## Conclusions

The current study looked into the practicability of EBT in a resource-limited setting and compared exchange transfusion in the setting of artesunate being used as a primary antimalarial. Though EBT causes a rapid drop in parasitaemia in the first six hours compared to antimalarial alone, this did not translate into a statistically significant survival benefit (p>0.05). However, a similar mortality in the EBT group (having sicker patients and a higher PI) compared with the chemotherapy-only group (having less sick patients with a lesser PI) indicates some advantage of EBT in very sick patients. Though the unavailability of enough fresh whole blood may hamper the efficacy of EBT in resource-limited areas, the current study demonstrates that blood volumes less than the standard recommendation of 70 ml/kg can help to effectively bring down the PI. It may also be worthwhile to accumulate more data comparing EBT against artesunate instead of quinine. Artesunate, by virtue of its rapid action, may be capable of questioning the very place of blood transfusion in complicated malaria. However, for this, one would require more randomised prospective trials.

## References

[1] (2023). Malaria. 2010.

[2] Chang CY (2023). Clinical characteristics and outcome of severe malaria in Kapit, Sarawak, Malaysian Borneo. J Vector Borne Dis.

[3] Gyr K, Speck B, Ritz R, Cornu P, Buckner CD (1974). Cerebral tropical malaria with blackwater fever. A current diagnostic and therapeutic problem (Article in German). Schweiz Med Wochenschr.

[4] Beards SC, Joynt GM, Lipman J (1994). Haemodynamic and oxygen transport response during exchange transfusion for severe falciparum malaria. Postgrad Med J.

[5] Salord F, Peyron F, Vuillez JP, Gaussorgues P (1991). TNF levels in severe malaria treated by exchange transfusion. Intensive Care Med.

[6] Cook GC (1991). Exchange blood transfusion for severe Plasmodium falciparum infection: proven adjunct in management or still sub judice?. J Infect.

[7] Panosian CB (1998). Exchange blood transfusion in severe falciparum malaria--the debate goes on. Clin Infect Dis.

[8] Pinanong M (1997). Exchange transfusion therapy in severe complicated malaria. J Med Assoc Thai.

[9] Srichaikul T, Leelasiri A, Polvicha P, Mongkonsritragoon W, Prayoonwiwat W, Leelarsupasri S, Puetpol S (1993). Exchange transfusion therapy in severe complicated malaria. Southeast Asian J Trop Med Public Health.

[10] Hoontrakoon S, Suputtamongkol Y (1998). Exchange transfusion as an adjunct to the treatment of severe falciparum malaria. Trop Med Int Health.

[11] Looareesuwan S, Phillips RE, Karbwang J, White NJ, Flegg PJ, Warrell DA (1990). Plasmodium falciparum hyperparasitaemia: use of exchange transfusion in seven patients and a review of the literature. Q J Med.

[12] Gulprasutdilog S, Chongkolwatana V, Buranakitjaroen P, Jaroonvesama N (1999). Exchange transfusion in severe falciparum malaria. J Med Assoc Thai.

[13] Boctor FN (2005). Red blood cell exchange transfusion as an adjunct treatment for severe pediatric falciparum malaria, using automated or manual procedures. Pediatrics.

[14] Shanbag P, Juvekar M, More V, Vaidya M (2006). Exchange transfusion in children with severe falciparum malaria and heavy parasitaemia. Ann Trop Paediatr.

[15] Alfandari S, Dixmier G, Guery B, Leroy O, Georges H, Beaucaire G (2001). Exchange transfusion for severe malaria. Infection.

[16] Deshpande A, Kalgutkar S, Udani S (2003). Red cell exchange using cell separator (therapeutic erythrocytapheresis) in two children with acute severe malaria. J Assoc Physicians India.

[17] Nieuwenhuis JA, Meertens JH, Zijlstra JG, Ligtenberg JJ, Tulleken JE, van der Werf TS (2006). Automated erythrocytapheresis in severe falciparum malaria: a critical appraisal. Acta Trop.

[18] Macallan DC, Pocock M, Robinson GT, Parker-Williams J, Bevan DH (2000). Red cell exchange, erythrocytapheresis, in the treatment of malaria with high parasitaemia in returning travellers. Trans R Soc Trop Med Hyg.

[19] Mordmüller B, Kremsner PG (1998). Hyperparasitemia and blood exchange transfusion for treatment of children with falciparum malaria. Clin Infect Dis.

[20] Reisinger EC, Silly HL, Smolle KH, Krejs GJ (1992). Exchange transfusion for severe falciparum malaria?. Infection.

[21] Burchard GD, Kröger J, Knobloch J (1997). Exchange blood transfusion in severe falciparum malaria: retrospective evaluation of 61 patients treated with, compared to 63 patients treated without, exchange transfusion. Trop Med Int Health.

[22] Riddle MS, Jackson JL, Sanders JW, Blazes DL (2002). Exchange transfusion as an adjunct therapy in severe Plasmodium falciparum malaria: a meta-analysis. Clin Infect Dis.

